# Investigation and management of vitamin D insufficiency and deficiency in acute adult psychiatric admissions: a clinical audit

**DOI:** 10.1192/bjo.2021.302

**Published:** 2021-06-18

**Authors:** Daniel Romeu, Rhiya Sood, Richard Hughes, Tariq Mahmood, Alastair Cardno

**Affiliations:** 1 Leeds and York Partnership NHS Foundation Trust, Leeds Institute of Health Sciences, University of Leeds; 2 School of Medicine, University of Leeds; 3 Leeds and York Partnership NHS Foundation Trust; 4 Leeds Institute of Health Sciences, University of Leeds

## Abstract

**Aims:**

Growing evidence suggests vitamin D as a contributing factor in psychiatric illness, particularly depression. Leeds and York Partnership NHS Foundation Trust (LYPFT) has a policy recommending that vitamin D levels are checked in all inpatients. The principal aims of this audit were to establish whether vitamin D levels were checked in inpatients and whether oral supplementation was commenced where appropriate, with a pre-determined target of 90% for both. The secondary aims were to assess whether rates of checking and replacing vitamin D, and mean vitamin D levels, differed between Caucasian and non-Caucasian populations.

**Method:**

We investigated adults aged 18–65 years newly admitted to the Becklin Centre, an acute psychiatric inpatient unit of four wards, between 1st December 2019 and 29th February 2020. 140 patients met eligibility criteria and were included in this study, of which 86 (61.4%) were Caucasian. Data were collected between 25th and 28th February 2021 by retrospectively reviewing two electronic patient record systems, Care Director and PPM, and the electronic prescribing platform EPMA. Results were compiled on a pre-determined data collection tool and analysed using Microsoft Excel. We defined insufficiency as serum 25-hydroxyvitamin D levels below 75nmol/l and deficiency as below 30nmol/l.

**Result:**

Vitamin D levels were checked in 79 (56.4%) inpatients, and the proportion checked differed significantly according to ethnicity (Caucasian = 64.0%, non-Caucasian = 44.4%; χ2 = 4.59, p = 0.032). Of these, 1 (1.3%) had an insufficient sample, 5 (6.3%) had normal levels, 41 (51.9%) had insufficient levels and 32 (40.5%) were deficient. Colecalciferol was commenced for 61 (83.6%) of those with insufficient or deficient vitamin D levels. Rates of colecalciferol prescribing did not differ between ethnic groups (Caucasian = 82.0%, non-Caucasian = 85.0%; χ2 = 0.091, p = 0.76). Mean vitamin D levels did not significantly differ (p = 0.77) between Caucasians (38.3nmol/l) and non-Caucasians (36.2nmol/l).

**Conclusion:**

LYPFT did not meet the target for testing for and treating vitamin D insufficiency and deficiency in psychiatric inpatients. Other blood results were often available when vitamin D levels were not, suggesting a lack of awareness of the guidance. Ethnicity influenced rates of vitamin D analysis but not replacement or mean serum levels. We aim to present our findings to the Trust's medical workforce to raise awareness of the relevant guidance. Given the paucity of psychiatric inpatients with normal vitamin D levels, further research into the role of vitamin D in psychopathology is warranted.

